# Effects of Dietary Vitamin C on the Growth Performance, Biochemical Parameters, and Antioxidant Activity of Coho Salmon *Oncorhynchus kisutch* (Walbaum, 1792) Postsmolts

**DOI:** 10.1155/2022/6866578

**Published:** 2022-12-26

**Authors:** Cong-mei Xu, Hai-rui Yu, Ling-yao Li, Min Li, Xiang-yi Qiu, Xiao-qian Fan, Yan-lin Fan, Ling-ling Shan

**Affiliations:** ^1^Key Laboratory of Biochemistry and Molecular Biology in Universities of Shandong (Weifang University), Weifang Key Laboratory of Coho Salmon Culturing Facility Engineering, Institute of Modern Facility Fisheries, College of Biology and Oceanography, Weifang University, Weifang 261061, China; ^2^Shandong Collaborative Innovation Center of Coho Salmon Health Culture Engineering Technology, Shandong Conqueren Marine Technology Co., Ltd., Weifang 261108, China

## Abstract

Vitamin C (VC) plays an essential role in fish physiological function and normal growth. However, its effects and requirement of coho salmon *Oncorhynchus kisutch* (Walbaum, 1792) are still unknown. Based on the influences on growth, serum biochemical parameters, and antioxidative ability, an assessment of dietary VC requirement for coho salmon postsmolts (183.19 ± 1.91 g) was conducted with a ten-week feeding trial. Seven isonitrogenous (45.66% protein) and isolipidic (10.76% lipid) diets were formulated to include graded VC concentrations of 1.8, 10.9, 50.8, 100.5, 197.3, 293.8, and 586.7 mg/kg, respectively. Results showed that VC markedly improved the growth performance indexes and liver VC concentration, enhanced the hepatic and serum antioxidant activities, and increased the contents of serum alkaline phosphatase (AKP) activity, low-density lipoprotein cholesterol (LDL-C), high-density lipoprotein cholesterol (HDL-C), and total cholesterol (TC) whereas decreased the serum aspartate aminotransferase (AST), alanine aminotransferase (ALT) activities, and triglyceride (TG) level. Polynomial analysis showed that the optimal VC levels in the diet of coho salmon postsmolts were 188.10, 190.68, 224.68, 132.83, 156.57, 170.12, 171.00, 185.50, 142.77, and 93.08 mg/kg on the basis of specific growth rate (SGR), feed conversion ratio (FCR), liver VC concentration, catalase (CAT), hepatic superoxide dismutase (SOD) activities, malondialdehyde (MDA) content, and serum total antioxidative capacity (T-AOC), AKP, AST, and ALT activities, respectively. The dietary VC requirement was in the range of 93.08–224.68 mg/kg for optimum growth performance, serum enzyme activities, and antioxidant capacity of coho salmon postsmolts.

## 1. Introduction

Fish require vitamins to survive because they act as enzyme cofactors [1], through which vitamins help organisms maintain optimal health and normal metabolic functions [2, 3]. As a water-soluble vitamin, vitamin C (VC) plays a crucial role in maintaining normal fish growth and physiological function [4–9]. Examples of physiological effects of VC proved in fish are related to reproduction [6, 10, 11], normal growth [12, 13], cartilage and bone formation [14], the iron metabolism and hematology [15, 16], lipid metabolism [17, 18], stress [19–21], immune response [22–24], and interactions with other micronutrients [25, 26]. VC had also been proved to reduce the oxidative stress of fish, thus benefiting the fish health [20, 27–29]. Compared to feeding with VC-deficient diet, yellow catfish, *Pelteobagrus fulvidraco* (Richardson, 1846), feeding with adequate dietary VC showed higher catalase (CAT), superoxide dismutase (SOD), and glutathione peroxidase (GPx) activities [30]. VC was also found to have an active influence on antioxidant ability of black carp, *Mylopharyngodon piceus* (Richardson, 1846) [31], and Siberian sturgeon, *Acipenser baerii* (Brandt, 1869) [32]. Thus, VC supplementation is required for fishes [1].

VC or L-ascorbic acid cannot be synthesized by teleost fishes, because of the mutation of the L-gulonolactone oxidase gene that encodes the enzyme charging for catalyzing the final procedure of VC de novo synthesis [33, 34]. Therefore, farmed fish need to gain VC through the diets for optimum growth and other physiological function maintenance. Inappropriate supplementation of VC would lead to lower enzyme activities, thus resulting in poor growth performance, low survival rate, and being susceptible to diseases of fish [1, 8, 30, 35]. Dietary VC deficiency would also lead to some nonspecific signs, such as changes of serum triglycerides and cholesterol levels [1, 36]. The dietary VC requirement had been investigated in many farmed fish species, and the optimal dietary VC levels are varied among fish species [1], which can be affected by the growth rates, age, size, various environmental factors, and nutrient interrelationships of the fish [1, 7, 24]. Consequently, it is crucial to analyze dietary VC requirement for a certain fish species.

In order to meet the increased needs for fish as food source, the aquaculture industry has dramatically expanded in recent years compared with other sectors of food production [37]. Coho salmon, *Oncorhynchus kisutch* (Walbaum, 1792), is a Pacific salmon (genus *Oncorhynchus*) species well known for its high protein and high unsaturated fatty acids (HUFAs) contents, especially omega-3 HUFAs which had a series of health benefits for human being [38–40]. Over the past few years, coho salmon is increasingly farmed in China. As large-scale farming expands, studies on the nutrition of coho salmon have been relatively backwards, and little is known about its dietary VC requirements for this fish. In this study, we investigated the efficacy of graded dietary VC on growth, serum biochemical parameters, and antioxidant activities of coho salmon postsmolts.

## 2. Materials and Methods

### 2.1. Diet Preparation

In Table 1, we present the formulation and approximate composition of diets. Seven isonitrogenous (45.66% protein) and isolipidic (10.76% lipid) diets were formulated. Ascorbic acid-2-phosphate (NHU Co. Ltd., Shaoxing, China) was added to the seven diets as a VC source because of its high heat resistance compared with the unprotected VC [41]. The corresponding levels of dietary VC were determined by high performance liquid chromatography (HPLC; LC-20AD, Shimadzu, Japan) to contain 1.8, 10.9, 50.8, 100.5, 197.3, 293.8, and 586.7 mg/kg, respectively (Table 1).

Vitamin mixtures and mineral mixtures were premixed with *α*-cellulose in advance. The dry ingredients, except vitamin and mineral mixtures, were sieved through a 60-mesh sieve and then weighed and mixed accurately in a Hobart-type mixer until homogenous; after that, vitamin and mineral mixtures were added and continued to mix. Finally, fish oil as well as distilled water (30%) was added in order; after that, the mixture was pelleted (diameters 3 mm) by using a laboratory pelleting mill. In order to ensure the quality of the diets, they were air-dried until moisture levels are less than 10% and sieved, after which they were sealed in airtight bags and stored at -20°C until used.

### 2.2. Fish and Experimental Procedures

The postsmolts were acclimated in the culture system with two weeks, during which they were fed with basal diets (with no VC supplementation). After the acclimation, 210 fish (initial body weight: 183.19 ± 1.91 g) were assigned randomly to 21 cages (water volume 1,000 l) in a pond. Fish were fed three times daily for 10 weeks, 4 weeks with 5% of body weight per day and the next 6 weeks with 3%. Food residues, if any, should be sucked out and collected, and then, the dry weight is determined by drying at 105°C. During the experimental period, fish were gently moved out and bulk weighed biweekly and were fasted at that very day. A high-pressure water gun was used to brush and rinse cages at the same time. The water flow rate was in the range of 1.8-2.2 l/min; the water pH and temperature were 7.4 ± 1.3 and 15.0 ± 1.5°C, respectively; dissolved oxygen was ranged between 7.5 and 8.0 mg O_2_/l; the ammonia nitrogen was 0.026 ± 0.01 mg/l; and the alkalinity was between 90-130 mg/l. Postsmolts were reared under natural lighting and in freshwater.

### 2.3. Sample Collection

Fish were counted and weighed in bulk from each cage after a 24-hour fast at the end of the feeding period, following which seven fish from each cage were sampled for analysis. For determining morphological indexes, containing hepatosomatic index (HSI), intestosomatic index (ISI), and condition factor (CF), three were selected from each cage. Following the measurement of the body length, another four were chose to gain serum samples by taking blood from the caudal vein. The blood samples stood for 2 hours at room temperature and then were centrifuged for 3500 g for 10 minutes; after that, the supernatant was collected and then stored at -80°C until it was analyzed for biochemical parameters and antioxidant activity. The 4 fish were dissected quickly after exsanguination, and the liver and muscle were removed and stored at -80°C. The liver samples were used to analyze hepatic VC concentration and antioxidant capacity, and muscle samples were for the assessments of muscle proximate composition.

### 2.4. Data Collection

#### 2.4.1. Growth Performance

The parameters of growth performance and body indices were calculated with the following formula:

(1) $$\begin{array}{c} \text{Survival rate }\left(\%\right)=100\times \frac{\text{final number of fish}}{\text{initial number of fish}},\\ \text{SGR }\left(\%/\text{day}\right)=100\times \frac{\ln\;\left(\text{final body weight},\text{g}\right)–\ln\;\left(\text{initial body weight},\text{g}\right)}{\text{days}},\\ \text{Feed conversion ratio }\left(\text{FCR},\%\right)=\frac{\text{dry feed intake }\left(\text{g}\right)}{\text{final body weight }\left(\text{g}\right)-\text{initial body weight }\left(\text{g}\right)},\\ \text{CF }\left(\%\right)=100\times \frac{\text{body weight }\left(\text{g}\right)}{\text{body lengt}{\text{h}}^{3}\left({\text{cm}}^{3}\right)},\\ \text{HSI }\left(\%\right)=100\times \frac{\text{liver weight }\left(\text{g}\right)\;}{\text{body weight }\left(\text{g}\right)},\\ \text{ISI }\left(\%\right)=100\times \frac{\text{intestinal weight }\left(\text{g}\right)}{\text{body weight }\left(\text{g}\right)}. \end{array}$$

#### 2.4.2. Proximate Composition Analysis

The moisture was assessed according to drying at 105°C, the ash was determined through incineration at 550°C, the crude lipid was extracted with petroleum ether and determined by the Soxhlet apparatus, and crude protein was determined by the Kjeldahl apparatus (nitrogen ×6.25) [42].

#### 2.4.3. Serum and Hepatic Biochemical Analysis

The liver samples were homogenized in 0.1 M Tris-HCl buffer (pH = 7.4) at 4°C; after that, the homogenate was gathered and then stored at -80°C until analyzed. The hepatic malondialdehyde (MDA) content; SOD and CAT activities; the serum aspartate aminotransferase (AST), alanine aminotransferase (ALT), alkaline phosphatase (AKP), and total antioxidative capacity (T-AOC) activities; high-density lipoprotein cholesterol (HDL-C), low-density lipoprotein cholesterol (LDL-C), triglyceride (TG), and total cholesterol (TC) contents were determined using the reagent kits (Nanjing Jiancheng Bioengineering Institute, Nanjing, China).

#### 2.4.4. VC Content Assay

The VC concentration of the experimental diets was analyzed by HPLC with a C18 column (5 *μ*m, 150 × 4.6 mm). The protocols were referred to a national standard method (GB/T238882-2009). In brief, the phosphate-buffered saline (PBS) solution was prepared to contain 0.054 g/ml KH_2_PO_4_; the mobile phase was prepared by dissolving the KH_2_PO_4_, tetrabutyl ammonium hydrogen sulfate, and methanol with a certain proportion; and then, the solution was filtered and degassed. The diet samples (about 3 g) were extracted in PBS, then degassed, centrifuged, and filtered. The tris-(cyclohexyl-ammonium)-ascorbic acid-2-phosphate (Merck, Germany) dissolving in PBS was used as standard sample. Hepatic VC content was analyzed using the reagent kit (Nanjing Jiancheng Bioengineering Institute, Jiangsu, China) [43].

#### 2.4.5. Statistical Analyses

The data are showed with means ± standard errors (SE). The response of the dependent variable to the VC level was described by orthogonal polynomial contrasts [44]. The means of each group were determined by Duncan's test, and *P* < .05 were considered to have significant difference. Linear and cubic regression analyses were used to analyze optimal dietary VC levels of coho salmon.

## 3. Results

### 3.1. Growth Performance

**Figure 1:**
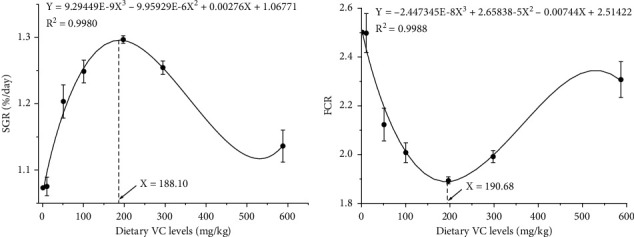
Cubic regression analysis of SGR with dietary vitamin C (VC) levels in coho salmon *Oncorhynchus kisutch* (Walbaum, 1792) postsmolts. The predicted dietary VC requirements of SGR and FCR are 188.10 and 190.68 mg/kg, respectively. Abbreviation: SGR: specific growth rate; FCR: feed conversion ratio.

Graded dietary VC concentrations did not have significant influence on the coho salmon postsmolts survival (*P* > .05) (Table 2). The SGR was increased and FCR decreased in fish when fed diets with graded VC concentrations (linear, quadratic, and cubic; *P* < .05). HSI, VSI, and CF showed no significant effect with different VC levels (*P* > .05). According to the polynomial regression analysis of SGR and FCR, the VC requirements for coho salmon postsmolts were predicted to be 188.10 and 190.68 mg/kg, respectively (Figures 1(a) and 1(b)).

### 3.2. Muscle Proximate Composition and Liver VC Concentration

**Figure 2:**
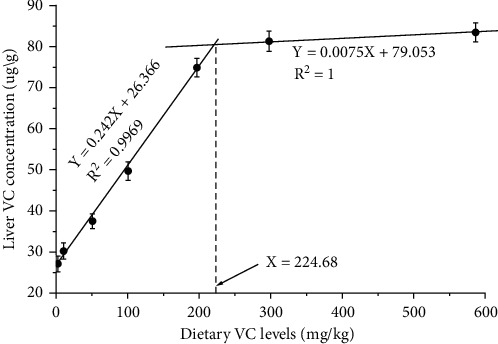
Broken-line analyses of liver vitamin C (VC) concentrations with dietary VC levels in coho salmon *Oncorhynchus kisutch* (Walbaum, 1792) postsmolts. The predicted dietary VC requirement based on liver VC concentration is 224.68 mg/kg.

Dietary VC levels did not have significant effects on the muscle proximate composition including moisture, crude protein, crude lipid, and ash of coho salmon postsmolts (*P* > .05) (Table 3). The crude protein content varied among 19.56 to 20.12%. The crude lipid content varied among 3.78 to 3.94%. The ash was ranged between 2.50 and 2.76%. The moisture was in the range of 71.59 to 73.54%. With the rising of VC concentrations in the diets, the hepatic VC concentration showed linear and cubic increases (*P* < .05) (Table 3). The optimal dietary VC requirement based on liver VC concentration was 224.68 mg/kg (Figure 2).

### 3.3. Biochemical Parameters and Enzymes Activities of Serum

**Figure 3:**
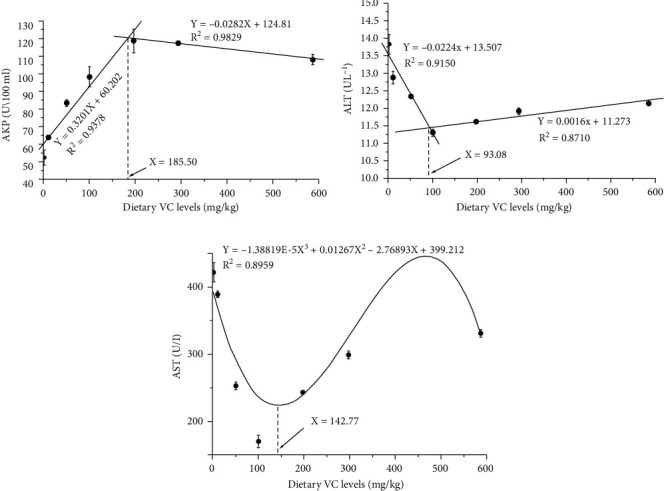
Broken-line analyses of AKP and ALT and cubic regression analysis of AST with dietary vitamin C (VC) levels in coho salmon *Oncorhynchus kisutch* (Walbaum, 1792) postsmolts. The predicted dietary VC requirements of AKP, ALT, and AST are 185.50, 93.08, and 142.77 mg/kg, respectively. Abbreviation: AKP: alkaline phosphatase; ALT: glutamic pyruvic transaminase; AST: glutamic oxaloacetic transaminase.

The TG level declined, and the LDL-C and HDL-C levels rose in linear, quadratic, and cubic manners when fish fed diets with increasing VC concentrations (*P* < .05). TC level was showed with linear and cubic rising trends (linear and cubic, *P* < .01) and the AKP activity with linear, quadratic, and cubic rising trends (*P* < .05) when fish fed diets with graded VC concentrations. With the VC concentrations increased, the AST activity was showed with linear, quadratic, and cubic reducing trends (*P* < .05) and ALT activity with linear and quadratic reducing trends (*P* < .05) (Table 4). The VC requirements for coho salmon postsmolts were 185.50, 142.77, and 93.08 mg/kg based on serum AKP, AST, and ALT activities, respectively (Figures 3(a)–3(c)).

### 3.4. Activities of Serum and Hepatic Antioxidant Enzymes

**Figure 4:**
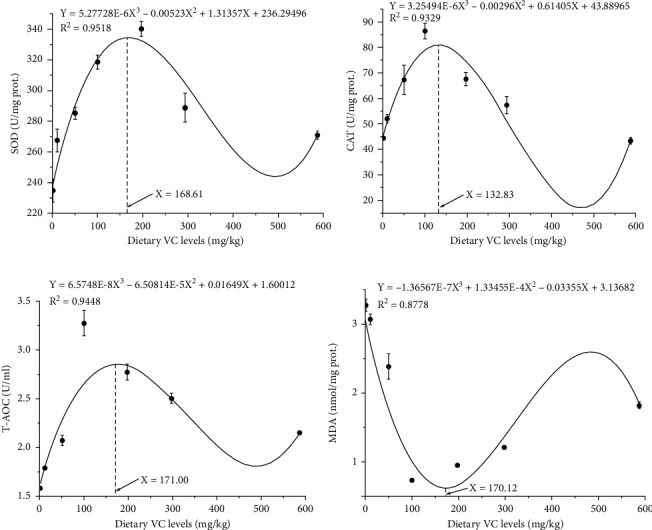
Cubic regression analyses of SOD, CAT activities, MDA concentration, and serum T-AOC activity with dietary vitamin C (VC) levels in coho salmon *Oncorhynchus kisutch* (Walbaum, 1792) postsmolts. The predicted dietary VC requirements of SOD, CAT, T-AOC, and MDA are 156.57, 132.83, 171.00, and 170.12 mg/kg, respectively. Abbreviation: SOD: superoxide dismutase; CAT: catalase; T-AOC: total antioxidative capacity; MDA: malondialdehyde.

The hepatic CAT, SOD activities, and serum T-AOC activity were risen, and the hepatic MDA contents declined in linear, quadratic, and cubic manners when fish fed diets with graded VC concentrations (*P* < .05) (Table 5). The VC requirements for coho salmon postsmolts were 71.46 and 176.19 mg/kg according to hepatic SOD, CAT activities (Figures 4(a) and 4(b)), MDA content (Figure 4(c)), and serum T-AOC activity (Figure 4(d)), respectively.

## 4. Discussion

It has been well known that dietary VC can enhance survival rates, growth, immune, and stress resistance [1, 7, 8, 30, 45]. Deficiency in VC supplementation caused retarded growth, poor survival rate, or abnormal pigmentation in many studied fishes [4, 24, 46, 47]. In the study, the adequate VC level of the diet could obviously improve the growth performance of coho salmon postsmolts. It was suggested that the improvement of growth might result from the rising of feed efficiency to the diet [7, 8]. VC might have effects on nutrient utilization, because of its importance in the process of protein metabolism and stimulating protein synthesis [48]. Dietary VC requirement was also needed for Atlantic salmon, *Salmo salar* (Linnaeus, 1758) alevins, in which the SGR was significantly improved with VC supplementation [9]. However, in rainbow trout, *Oncorhynchus mykiss* (Walbaum, 1792), the growth was not obviously changed when fed with diets with graded VC levels [49]. Impacts of VC supplementation on fish can be influenced by fish species, size, developmental stage, and variation in experimental conditions as well as cultivation environment [1].

This study showed that no other VC deficiency signs mentioned above were observed throughout the experiment except a reduction in weight gain. This was similar with that in yellow croaker *Pseudosciaena amblyceps* (Richardson, 1846) [50], Atlantic salmon [51], and seabream *Sparus aurata* (Linnaeus, 1758) [52]. Lack of phenotype might be caused by the fish size in the experiment, which was larger than that of Japanese seabass *Lateolabrax japonicus* (Cuvier, 1828) [12], common carp *Cyprinus carpio* (Linnaeus, 1758) [53], and hybrid tilapia *Oreochromis niloticus* × *Oreochromis aureus* [54]. According to a study about the effects of size or age on VC supplement deficiency in fish, the size of fish was positively related to the onset of fish deficiency symptoms [55].

TG and TC are suggested to be important in the process of lipid metabolism. High TG levels can result in liver dysfunction, nephritic syndrome, and glycogen storage disease [56, 57]. In this study, VC supplementation obviously decreased the TG level of serum, suggesting that dietary VC levels did affect the coho salmon health. Blood TC including HDL-C as well as LDL-C levels had been documented to decrease [58–61], increase [36, 62, 63], or be independent [23, 30, 64] with dietary VC supplementation in fishes. VC had synergistic effects on cholesterol synthesis and catabolism; this might be the reason that VC had variable influences on serum cholesterol contents [65]. In the study, TC, LDL-C, and HDL-C contents showed dramatic increase with VC supplementation, which were similar with those in rainbow trout [36]. It was suggested that dietary VC levels increased the cholesterol synthesis and inhibited the degradation and clearance of serum LDL-C [36].

Radicals like reactive oxygen species (ROS) were considered to be harmful to some vital cellular components, for example, DNA, proteins, carbohydrates, and lipids [35, 66]. The increasing of free radical production in cells would lead to oxidative stress and further result in cell and tissue damages [67, 68]. MDA, produced by lipid peroxidation, is toxic for cells, whose structure and function would be damaged. As a consequence, MDA is suggested to be an indicator of oxidative cell damage [69]. The antioxidative enzymes like CAT and SOD could eliminate excess ROS, thus decreased the damages caused by lipid peroxidation [70, 71]. In the present study, fish supplied with appropriate dietary VC exhibited higher contents of serum T-AOC and hepatic CAT, SOD, and lower levels of hepatic MDA. This was in agreement with that in the studies of thornfish *Bovichtus variegatus* (Richardson, 1846) [72], juvenile cobia [24], juvenile black carp *Mylopharyngodon piceus* (Richardson, 1846) [32], and Nile tilapia fingerlings [8]. In addition, the AKP can act as a hydrolytic enzyme which could promote the recognition and phagocytosis capacity of organism, thus increased the nonspecific immunity of organism [73]. Serum AKP levels in cobia, *Rachycentron canadum* (Linnaeus, 1766), and Tilapia, *Oreochromis niloticus*, (Linnaeus, 1758) were significantly increased with VC supplementation [24, 74]. Another previous study showed that the serum AKP level was increased with VC supplementation in Wuchang bream, *Megalobrama amblycephala* (Yih, 1955), when suffered with stress from pH [46]. This study also reported that the serum AKP activity was increased with adequate VC supplementation of coho salmon. All these are considered to be attributed to the powerful antioxidative activity of VC. Acting as an electron donor, VC is an antioxidant that scavenges free radicals, thus inhibiting radical injuries to cellular components [75, 76].

ALT and AST generally exist in the body [77]. These enzymes also play key roles in evaluating the healthy status of the liver and some other organs [78]. Their higher activities in serum may suggest organ dysfunction or tissue injury [79]. AST and ALT are important in hepatic function that contributes to the mutual transformation of amino acid, carbohydrates, and lipid. Their contents are high in tissue and low in serum when fish is in health status [35]. The rising of AST and ALT activities in serum might primarily be caused by hepatic AST and ALT enzymes leaking to the extracellular space and ultimately to the plasma [80], indicating the hepatotoxic effect of VC deficiency. In this report, the ALT and AST levels exhibited obvious declines following treatment with adequate VC supplementation, suggesting the efficacy of this nutrient to protect liver cells from damages. This is in agreement with that in juvenile Chinese sucker *Myxocyprinus asiaticus* (Bleeker, 1865) and juvenile striped catfish *Pangasianodon hypophthalmus* (Sauvage, 1878), in which the optimal dietary VC is helpful to maintain the proper function of liver [35, 81].

## 5. Conclusion

In conclusion, adequate VC supplementation is essential to improve the growth performance and liver VC content; decreased serum TG level and AST and ALT activities; increased serum TC, HDL-C, and LDL-C contents; and enhanced antioxidant capacity of coho salmon postsmolts. The optimal VC level was in the range of 93.08–224.68 mg/kg for optimum growth performance, serum enzyme activities, and antioxidative ability of coho salmon postsmolts.

## Figures and Tables

**Table 1:** Formulation and proximate composition of the experimental diets for coho salmon *Oncorhynchus kisutch* (Walbaum, 1792) postsmolts (% dry matter).

Ingredients	Dietary vitamin C levels (mg/kg)						
	1.8	10.9	50.8	100.5	197.3	293.8	586.7
Casein^1^	38.0	38.0	38.0	38.0	38.0	38.0	38.0
Gelatin	12.0	12.0	12.0	12.0	12.0	12.0	12.0
Corn oil	6.0	6.0	6.0	6.0	6.0	6.0	6.0
Soybean oil^1^	3.0	3.0	3.0	3.0	3.0	3.0	3.0
Dextrin	28.0	28.0	28.0	28.0	28.0	28.0	28.0
*α*-Cellulose^1^	8.0	8.0	8.0	8.0	8.0	8.0	8.0
Mineral premix^2^	2.5	2.5	2.5	2.5	2.5	2.5	2.5
Vitamin premix-vitamin C free^3^	1.0	1.0	1.0	1.0	1.0	1.0	1.0
Ca(H_2_PO_4_)_2_	1.5	1.5	1.5	1.5	1.5	1.5	1.5
L-ascorbic acid-2-phosphate (mg/kg)	0	30	150	300	600	900	1800
Proximate composition							
Moisture (%)	11.16	11.24	11.18	11.26	10.96	11.21	11.12
Crude protein (%)	45.87	45.46	45.58	45.51	45.63	45.79	45.75
Crude lipid (%)	10.73	10.70	10.81	10.79	10.75	10.81	10.73
Ash (%)	5.37	5.42	5.41	5.53	5.45	5.63	5.78
Vitamin C (mg/kg)	1.8	10.9	50.8	100.5	197.3	293.8	586.7

^1^Provided by Shandong Conqueren Marine Technology Co., Ltd., Weifang, China. ^2^Composition (mg kg^–1^ mineral premix): AlK (SO_4_)_2_·12H_2_O, 123.7; CaCl_2_, 17879.8; CuSO_4_·5H_2_O, 31.7; CoCl_2_·6H_2_O, 48.9; FeSO_4_·7H_2_O, 707.4; MgSO_4_·7H_2_O, 4316.8; MnSO_4_·4H_2_O, 31.1; ZnSO4·7H2O, 176.7; KCl, 1191.9; KI, 5.3; NaCl, 4934.5; Na_2_SeO_3_·H_2_O, 3.4; Ca (H_2_PO_4_)_2_·H_2_O, 12457.0; KH_2_PO_4,_ 9930.2. ^3^Vitamin premix supplied the diets with (mg/kg dry diet): cholecalciferol, 0.04; *α*-tocopherol, 50; menadione, 40.0; thiamine-HCl, 12.0; riboflavin, 25.0; D-calcium pantothenate, 20; pyridoxine-HCl, 15.0; choline chloride, 500.0; *meso*-inositol, 200.0; D-biotin, 0.5; folic acid, 1.5; retinal palmitate, 0.75; niacin, 75.0; cyanocobalamin, 0.01.

**Table 2:** Survival and growth performance and feed utilization of coho salmon *Oncorhynchus kisutch* (Walbaum, 1792) postsmolts fed the experimental diets with different vitamin C (VC) levels for 10 weeks^1^.

Dietary VC levels (mg/kg)	1.8	10.9	50.8	100.5	197.3	293.8	586.7	Regression analysis^2^		
								Linear	Quadratic	Cubic
SR (%)	93.33 ± 3.33	96.67 ± 3.33	96.67 ± 3.33	100	100	100	93.33 ± 6.67	0.58	0.09	0.43
IBW (g)	181.00 ± 1.00	182.33 ± 1.20	183.33 ± 0.67	184.33 ± 1.20	183.33 ± 0.88	184.33 ± 1.20	183.67 ± 1.20	0.35	0.18	0.80
FBW (g)	383.68 ± 2.70^a^	386.87 ± 1.32^a^	425.56 ± 6.71^c^	441.46 ± 2.95^d^	454.35 ± 3.95^d^	443.49 ± 2.58^d^	407.13 ± 9.22^b^	<0.01	<0.01	<0.01
SGR (%/day)	1.073 ± 0.002^a^	1.075 ± 0.014^a^	1.203 ± 0.025^c^	1.248 ± 0.017^cd^	1.297 ± 0.006^d^	1.254 ± 0.010^cd^	1.137 ± 0.024^b^	<0.01	<0.01	<0.01
FCR	2.501 ± 0.008^d^	2.497 ± 0.079^d^	2.123 ± 0.067^b^	2.009 ± 0.041^ab^	1.894 ± 0.014^a^	1.992 ± 0.023^ab^	2.307 ± 0.073^c^	<0.01	<0.01	<0.01
CF (g/cm^3^)	1.270 ± 0.162	1.111 ± 0.059	1.228 ± 0.008	1.272 ± 0.168	1.193 ± 0.043	1.409 ± 0.059	1.204 ± 0.062	0.496	0.983	0.192
HSI (%)	0.661 ± 0.061	0.750 ± 0.081	0.604 ± 0.016	0.656 ± 0.035	0.668 ± 0.029	0.714 ± 0.006	0.713 ± 0.028	0.530	0.299	0.825
ISI (%)	5.160 ± 0.633	5.294 ± 0.670	4.606 ± 0.431	4.557 ± 0.455	4.780 ± 0.231	4.933 ± 0.240	5.775 ± 0.120	0.588	0.06	0.471

Abbreviations: SR: survival rate; IBW: initial body weight; FBW: final body weight; FCR: feed conversion ratio; CF: condition factor; HSI: hepatosomatic index; ISI: intestosomatic index. ^1^Each value represents the mean of 3 replicates. ^2^Orthogonal polynomial contrasts were used to assess the significance of linear, quadratic, or cubic models to describe the response in the dependent variable to VC level. A probability value of *P* < .05 was described to be statistically significant.

**Table 3:** Muscle proximate composition and liver vitamin C (VC) concentrations of coho salmon *Oncorhynchus kisutch* (Walbaum, 1792) postsmolts fed the experimental diets with graded VC levels after 10 weeks^1^.

Dietary VC levels (mg/kg)	1.8	10.9	50.8	100.5	197.3	293.8	586.7	Regression analysis^2^		
								Linear	Quadratic	Cubic
Muscle										
Moisture (%)	73.14 ± 0.16	72.98 ± 0.60	71.69 ± 0.22	73.21 ± 0.36	73.11 ± 0.03	73.54 ± 0.16	73.32 ± 0.25	0.21	0.47	0.30
Ash (%)	2.50 ± 0.20	2.76 ± 0.08	2.69 ± 0.08	2.75 ± 0.15	2.53 ± 0.08	2.75 ± 0.06	2.76 ± 0.10	0.37	0.74	0.16
Crude protein (%)	19.96 ± 0.09	19.95 ± 0.60	20.04 ± 0.30	19.73 ± 0.26	20.12 ± 0.07	19.56 ± 0.16	19.74 ± 0.36	0.43	0.76	0.90
Crude lipid (%)	3.78 ± 0.03	3.83 ± 0.04	3.94 ± 0.05	3.93 ± 0.15	3.88 ± 0.04	3.81 ± 0.06	3.83 ± 0.09	0.91	0.11	0.38
Liver										
VC content (*μ*g/g)	27.06 ± 1.96^a^	30.22 ± 2.00^a^	37.47 ± 1.76^b^	49.62 ± 2.76^c^	74.89 ± 2.24^d^	81.28 ± 2.53^e^	83.44 ± 2.3^e^	<0.01	0.41	<0.01

^1^Each value represents the mean of 3 replicates. ^2^Orthogonal polynomial contrasts were used to assess the significance of linear, quadratic, or cubic models to describe the response in the dependent variable to VC level. A probability value of *P* < .05 was described to be statistically significant.

**Table 4:** Serum biochemical parameters of coho salmon *Oncorhynchus kisutch* (Walbaum, 1792) postsmolts fed the experimental diets with graded vitamin C (VC) levels after 10 weeks^1^.

Dietary VC levels (mg/kg)	1.8	10.9	50.8	100.5	197.3	293.8	586.7	Regression analysis^2^		
								Linear	Quadratic	Cubic
TG (mmol/l)	3.66 ± 0.26^d^	3.53 ± 0.13^d^	2.95 ± 0.06^c^	2.19 ± 0.01^b^	1.62 ± 0.07^a^	1.99 ± 0.07^b^	2.25 ± 0.08^b^	<0.01	<0.01	<0.01
TC (mmol/l)	8.29 ± 0.13^a^	8.65 ± 0.20^a^	9.60 ± 0.19^b^	9.60 ± 0.17^b^	10.64 ± 0.14^c^	11.98 ± 0.16^d^	11.46 ± 0.13^d^	<0.01	0.82	<0.01
LDL-C (mmol/l)	2.24 ± 0.10^a^	2.31 ± 0.13^a^	2.77 ± 0.10^bc^	3.00 ± 0.08^c^	3.32 ± 0.10^d^	3.05 ± 0.07^cd^	2.66 ± 0.10^b^	<0.01	<0.01	<0.01
HDL-C (mmol/l)	4.45 ± 0.15^a^	4.27 ± 0.12^a^	4.98 ± 0.10^b^	5.00 ± 0.07^b^	5.42 ± 0.13^b^	5.32 ± 0.16^b^	4.94 ± 0.27^b^	<0.01	<0.01	0.02
AKP (U/100 ml)	52.49 ± 4.33^a^	63.88 ± 0.63^a^	83.35 ± 1.84^b^	98.22 ± 5.46^c^	118.62 ± 6.57^d^	117.24 ± 0.22^d^	108.03 ± 2.89^cd^	<0.01	<0.01	<0.01
AST (U/l)	421.81 ± 14.19^e^	389.05 ± 4.83^de^	253.1 ± 5.7^b^	170.5 ± 8.91^a^	243.1 ± 1.44^b^	299.1 ± 5.61^c^	331.7 ± 4.62^d^	<0.01	<0.01	<0.01
ALT (U/l)	13.83 ± 0.28^e^	12.88 ± 0.18^d^	12.34 ± 0.02^c^	11.31 ± 0.10^a^	11.62 ± 0.04^ab^	11.92 ± 0.09^bc^	12.14 ± 0.02^c^	<0.01	<0.01	0.966

^1^Each value represents the mean of 3 replicates. ^2^Orthogonal polynomial contrasts were used to assess the significance of linear, quadratic, or cubic models to describe the response in the dependent variable to VC level. A probability value of *P* < .05 was described to be statistically significant. Abbreviations: TG: triglyceride; TC: total cholesterol; LDL-C: low-density lipoprotein cholesterol; HDL-C: high-density lipoprotein cholesterol; AKP: alkaline phosphatase; ALT: alanine aminotransferase; AST: aspartate aminotransferase.

**Table 5:** Serum and hepatic antioxidative enzyme activities in coho salmon *Oncorhynchus kisutch* (Walbaum, 1792) postsmolts fed the experimental diets with graded vitamin C (VC) levels after 10 weeks^1^.

Dietary VC levels (mg/kg)	1.8	10.9	50.8	100.5	197.3	293.8	586.7	Regression analysis^2^		
								Linear	Quadratic	Cubic
Liver										
SOD (U/mg prot.)	234.94 ± 7.36^a^	267.47 ± 7.53^b^	285.25 ± 3.96^b^	318.49 ± 4.48^c^	340.14 ± 4.71^c^	288.82 ± 9.36^b^	270.86 ± 2.69^b^	<0.01	<0.01	<0.01
CAT (U/mg prot.)	44.39 ± 0.86^a^	52.04 ± 1.65^ab^	67.28 ± 5.73^c^	86.39 ± 3.07^d^	67.56 ± 2.58^c^	57.36 ± 3.38^b^	43.38 ± 1.05^a^	<0.01	<0.01	<0.01
MDA (nmol/mg prot.)	3.27 ± 0.09^e^	3.07 ± 0.08^e^	2.38 ± 0.19^d^	0.73 ± 0.03^a^	0.95 ± 0.03^a^	1.21 ± 0.03^b^	1.82 ± 0.04^c^	<0.01	<0.01	<0.01
Serum										
T-AOC (U/ml)	1.58 ± 0.02^a^	1.79 ± 0.01^ab^	2.07 ± 0.05^bc^	3.27 ± 0.13^e^	2.77 ± 0.08^d^	2.50 ± 0.05^d^	2.15 ± 0.02^c^	<0.01	<0.01	<0.01

Abbreviations: CAT: catalase; MDA: malondialdehyde; SOD: superoxide dismutase; T-AOC: total antioxidant capacity. ^1^Each value represents the mean of 3 replicates. ^2^Orthogonal polynomial contrasts were used to assess the significance of linear, quadratic, or cubic models to describe the response in the dependent variable to VC level. A probability value of *P* < .05 was described to be statistically significant.

## Data Availability

All the data in the article are available from the corresponding author upon reasonable request.
